# Cinnarizine and dimenhydrinate in the treatment of vertigo in medical practice

**DOI:** 10.1007/s00508-015-0905-5

**Published:** 2015-12-11

**Authors:** Arne-Wulf Scholtz, Justus Ilgner, Benjamin Loader, Bernd W. Pritschow, Gerhard Weisshaar

**Affiliations:** 1 Department of Otorhinolaryngology, Division of Neurotology, Medical University of Innsbruck, Anichstrasse 35, 6020 Innsbruck, Austria; 2 Department of Oto-Laryngology and Plastic Head and Neck Surgery, University Hospital Aachen, Aachen, Germany; 3 Department of Otorhinolaryngology, Head and Neck Surgery, Rudolfstiftung Tertiary Teaching Hospital, Vienna, Austria; 4 Medical Department, Hennig Arzneimittel, Floersheim am Main, Germany

**Keywords:** Vertigo, Cinnarizine, Dimenhydrinate, Fixed combination, Noninterventional study

## Abstract

The efficacy and safety of the fixed combination of cinnarizine 20 mg and dimenhydrinate 40 mg in the treatment of vertigo of various origins have been investigated in a prospective, noninterventional study involving private practices throughout Germany. A total of 1275 patients with an average age of 61.2 years participated in the study. The vertigo symptoms, measured by a validated mean vertigo score (primary efficacy endpoint) improved by 61 % in the course of the observational period (median: 6 weeks). Concomitant symptoms frequently associated with vertigo such as nausea, vomiting and tinnitus were also markedly reduced by 84, 85 and 51 %, respectively. Overall efficacy has been rated by the physicians as ‘very much improved’ or ‘much improved’ in 95 % of the patients. A total of 47 patients (3.7 %) reported 51 adverse drug reactions (all nonserious). The results indicate a good tolerability and efficacy of the fixed combination of cinnarizine and dimenhydrinate in the treatment of vertigo in daily medical practice, which is in line with previous findings of numerous interventional, randomised, double-blind, controlled clinical trials.

## Introduction

Vertigo is one of the most common complaints reported in daily medical practice [1, 2], with a general prevalence of 17–32 %, among the population over 80 years up to 39 % [3]. The prevalence of vertigo increases with age [4, 5] resulting in a steady rise in incidence due to the global rise in general life expectancy. Especially in the elderly, vertigo is commonly associated with significant morbidity and even immobilisation or chronic invalidism, especially when aggravated by falls and resulting injury [6, 7]. In general, vertigo can impose considerable limitations on the patients’ ability to cope with daily activities, leading to a self-perceived reduction in the quality of life [4, 8] Therefore, the availability of an effective antivertigo treatment is of particular importance in medical practice, both to provide instant relief to the affected patients and for pharmacoeconomic reasons.

As vertigo may be associated with a wide range of underlying disorders, it constitutes a particular challenge in both primary and secondary care. In many cases, subjective vertigo symptoms can hardly be unambiguously attributed to a defined organic correlate, often leading to rather unspecified diagnoses. In particular, general practitioners (GPs) require an effective vertigo treatment option without having direct access to sophisticated diagnostic equipment needed for detailed diagnostics, especially in elderly patients with a high number of complex comorbidities.

The fixed combination of cinnarizine 20 mg and dimenhydrinate 40 mg (Arlevert^®^, Hennig Arzneimittel, Floersheim am Main, Germany) has been successfully used in the treatment of vertigo of various origins for more than three decades. Its efficacy is based on a dual mechanism of action, with the specific calcium channel blocker cinnarizine acting predominantly on the peripheral vestibular system and the antihistamine dimenhydrinate acting predominantly on the central vestibular system. The efficacy and safety of the combination preparation has been demonstrated in numerous randomised, double-blind, controlled clinical trials comprising a variety of patient populations [9–17]. The present noninterventional study was undertaken to determine whether the good efficacy and tolerability of the fixed-combination preparation reported in controlled clinical trials can be reproduced in routine clinical practice, which in turn provides deeper insight into the product’s effectiveness and safety when used in larger patient collectives.

## Patients, materials and methods

This prospective, open-label, noninterventional study has been carried out in primary and secondary care private practices throughout Germany between January 1992 and August 2008. Physicians, who prescribed the commercially available fixed-combination preparation for the treatment of vertigo patients in the course of normal clinical practice, have been offered to document the treatment outcome on standardised case report forms provided by the sponsor. Thus, prior to the inclusion of a patient in the study, it was at the participating physicians own discretion to use the combination preparation for the treatment of patients with vertigo of various origins in accordance with the terms of the summary of product characteristics (SmPC). For the selection of patients, no specific requirements were in place regarding inclusion or exclusion criteria, apart from the contraindications and safety recommendations listed in the product information. The physicians determined the starting dose (recommended dosage 3 × 1, up to 5 × 1 tablet per day), administration frequency and any later changes to either dose or frequency as well as the duration of treatment in line with the applicable German SmPC and independent of study documentation.

Demographic data, medical history, differential diagnosis and duration of vertigo, prior medical treatment, concomitant diseases and concomitant medications were registered on the occasion of the first visit. Patients rated the intensity of vertigo and concomitant symptoms (see below) by means of a 5-point verbal rating (or visual analogue) scale ranging from no vertigo (0) to slight (1), medium (2), strong (3) and very strong (4), both at the beginning (baseline) and at the end of the observational period. For approximately one third of the study participants, additional data were registered on the occasion of an interim visit after approximately 2 weeks. Furthermore, the physician assessed the overall (‘global’) efficacy by means of a 5-point verbal rating scale from very much improved (4) to much improved (3), slightly improved (2), not improved (1) and deteriorated (0).

The change in a validated MVS between baseline and end of observation has been used as the primary efficacy endpoint. The MVS was calculated as the mean of the following six (unprovoked) vertigo symptoms: dysstasia and walking unsteadiness, staggering, rotary sensation, tendency to fall, lift sensation and blackout. Secondary efficacy endpoints included the changes in concomitant symptoms (nausea, vomiting, sweating, tachycardia, tinnitus, impaired hearing) and the physicians’ global judgement of efficacy. A subgroup analysis was performed with respect to the type of vertigo.

The safety evaluation comprised the documentation of adverse events (AEs), including their classification with respect to seriousness, severity and possible relatedness to the study medication (adverse drug reaction, ADR). Additionally, the tolerability of the treatment was assessed by the physician on a 4-point verbal rating scale (very good, good, moderate or poor) on the occasion of the final visit.

Due to the observational character of the study, analyses were descriptive in nature. After double data entry, statistical analyses were performed using SAS 9.1.3 (SAS Institute Inc.). Assuming continuous but not necessarily normal distribution of the data, the Wilcoxon signed rank test was used to test the changes in the intensity of vertigo symptoms on the 5 % significance level. In order to account for possible deficiencies in the distributional properties of the MVS, as an alternative for parametric analysis (based on the mean), the median was calculated for baseline, final visit and for the changes between the two visits. For the median of the changes, nonparametric 95 % confidence intervals (CI95 %) based on order statistics are provided in addition to the parametric confidence intervals for the mean (based on the t-test model). Statistical analysis was performed using the intent-to-treat data set (safety population), including all patients who had been enrolled in the study. Missing data were imputed conservatively, that is values missing at baseline and at the end of observation were replaced by the best and the worst corresponding value of a patient in the sample, respectively. In addition, all analyses were performed on the basis of all available data without imputing missing values (‘data as available’) in order to check the robustness of the results.

## Results

Overall, 443 private practices throughout Germany participated in this observational study and contributed data from a total of 1275 patients. The physicians involved in the study mainly comprised GPs/internal medicine specialists, otorhinolaryngologists and neurologists/psychiatrists (Table 1). Male (34 %) and female (66 %) patients aged between 15 and 99 years (mean age: 61.2 years) were included in the study; female patients were on average slightly older than male patients (for details see Table 2). The mean duration of vertigo before enrolment in the study was approximately 1 year and ranged from less than 1 month to up to 20 years (Table 2). Nearly one third of the patients had taken antivertigo drugs before inclusion in the study. The most commonly used drugs were a homeopathic combination preparation, betahistine and *Ginkgo biloba* (Table 3). It can be assumed that in most cases the prior medical treatment had been discontinued because of insufficient effectiveness, and for this reason it was replaced by the combination preparation.

**Table 1:** Distribution of patients *(n* = 1275) among the medical disciplines participating in the study

Medical discipline	Number of patients	%
GPs/internal medicine specialists	729	57.2
Otorhinolaryngologists	419	32.9
Neurologists/psychiatrists	121	9.5
Others (e.g. surgeons)	6	0.4

**Table 2:** Selected demographic data and other baseline characteristics (safety population; *n* = 1275): number (*n*) and percentage (%) of patients, mean value of the respective parameter (Mean), standard deviation (SD) and range (minimum (Min), median (Med) maximum (Max))

Parameter	Patients		Statistical data				
	na	%	Mean	SD	Range		
					Min	Med	Max
Age *(years)*	1272		61.2	15.5	15	63	99
Male	433	34	59.4	14.7	15	61	93
Female	837	66	62.1	15.7	19	64	99
Height *(cm)*	1238		167.9	8.1	146	168	193
Weight (*kg)]*	1236		73.0	11.5	39	72	120
BMI *(kg/m* ^*2*^ *)*	1236		25.9	3.5	14.7	25.7	42.2
Duration of vertigo *(months)*	1189		12.3	23.1	< 1	3	240

^a^Data not available for all enrolled patients

**Table 3:** Antivertigo drug treatment before enrolment in the study: a total of 400 drugs were documented in 375 of 1275 patients (29.4 %); number *(n)* and percentage (%) of patients

Antivertigo drug	*n*	%
Homeopathic combination	114	28.50
Betahistine	95	23.75
*Ginkgo biloba*	69	17.25
Dimenhydrinate	43	10.75
Naphtidrofuryl	33	8.25
Sulpiride	27	6.75
Others (e.g. cinnarizine, meclizine)	19	4.75

For 799 patients (62.7 %), 1476 concomitant diseases have been documented, the most frequent of which were essential (primary) hypertension *(n* = 289), chronic ischaemic heart disease *(n* = 136), unspecified diabetes mellitus *(n* = 95) and disorders of lipoprotein metabolism and other lipidaemias *(n* = 71). A total of 603 patients (47.3 %) had received 1206 concomitant medications; ACE inhibitors (plain: *n* = 96, combinations: *n* = 21), beta blocking agents *(n* = 102), analgesics and antipyretics *(n* = 88), blood glucose lowering drugs excluding insulin *(n* = 87), vasodilators used in cardiac diseases *(n* = 83) and lipid-modifying agents *(n* = 61) were the most frequently prescribed drugs.

The vast majority of study participants had been prescribed either the standard dosage of 3 × 1 tablet per day of the fixed combination (89.1 % of the patients) or a reduced dosage of 2 × 1 tablet per day (8.6 %). For 110 patients, a change of the dosage in the course of the treatment has been reported, with a dose increase in 27 patients and a dose reduction in 83 patients. The average duration of treatment was 8.4 ± 1.5 weeks, with a broad range of between less than 1 week and 162 weeks (median: 6 weeks).

For 424 patients, additional data were registered on occasion of an interim visit after around 2 weeks of treatment. Only 21 patients (1.7 %) terminated the study prematurely, 7 because of complete remission of vertigo symptoms until the interim visit, 2 because of inadequate efficacy, 3 due to AEs and 9 for unknown reasons.

**Fig. 1:**
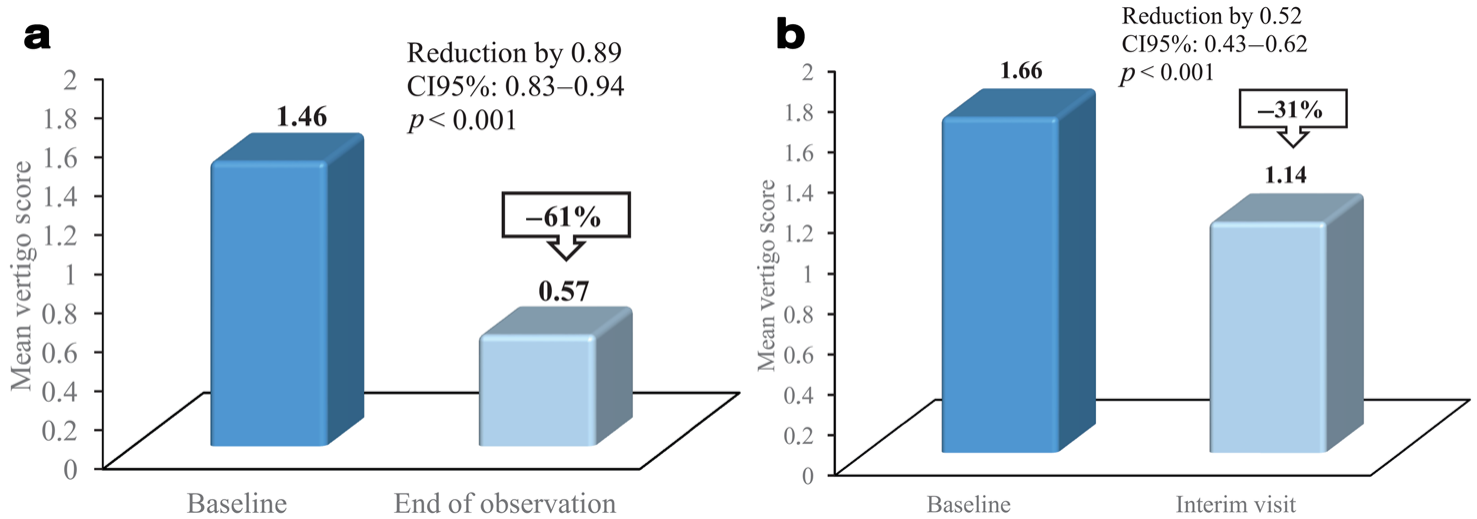
Reduction of the mean vertigo score (MVS (Mean score of six single, unprovoked vertigo symptoms (dysstasia and walking unsteadiness, staggering, rotary sensation, tendency to fall, lift sensation and blackout)) after treatment with a fixed-combination preparation of cinnarizine 20 mg and dimenhydrinate 40 mg. **a** Difference between baseline and end of observation (*n* = 1275). Vertigo symptoms improved significantly (*p* < 0.001). Approximately, 22% of the patients have been completely symptom-free at the end of observation. **b** Difference between baseline and interim visit (*n* = 424). Vertigo symptoms already improved significantly (*p* < 0.001) after approximately 2 weeks of treatment

Based on 1275 patients (safety population), treatment with the fixed-combination preparation led to a distinct improvement of the MVS in the course of the observational period. The mean value of the MVS was reduced from 1.46 at baseline to 0.57 at the end of observation, corresponding to a 61 % reduction by 0.89 (CI95 %: 0.83–0.94), whereas the median reduction from baseline was 0.93 (CI95 %: 0.83–1.00). The reduction was statistically significant (Wilcoxon signed rank test: *p*  < 0.001); for more details see Table 4 and Fig. 1a. A total of 282 patients (22.1 %) were completely symptom-free at the end of observation. Even after the relatively short period of treatment until the interim visit, a statistically significant mean reduction of the MVS by 0.52 (CI95 %: 0.43–0.62; *p* < 0.001) from 1.66 to 1.14 has been achieved (Fig. 1b), with a median reduction from baseline of 0.57 (CI95 %: 0.50–0.62).

**Table 4:** Reduction of the mean vertigo score (MVS) depending on type of vertigo and with respect to all patients: number *(n)* and percentage (%) of patients, mean value (Mean) with standard deviation (SD), median (Med) and 95 % confidence intervals (CI95 %)

	Patients		MVS				Reduction in MVS			
Type of vertigo			Baseline		Final visit					
	*n*	%	Mean	Med	Mean	Med	Mean	Med	CI95 %^a^	
			(SD)		(SD)		(SD)		Mean	Med
Peripheral vestibular	399	31.3	1.46 (0.73)	1.33	0.54 (0.82)	0.22	0.92 (1.13)	1.00	0.82–1.04	0.97–1.03
Central vestibular	113	8.9	1.38 (0.60)	1.33	0.50 (0.59)	0.33	0.88 (0.75)	0.83	0.73–1.02	0.67–1.00
Combined central-peripheral	261	20.5	1.59 (0.66)	1.52	0.74 (0.71)	0.58	0.85 (0.82)	0.83	0.75–0.95	0.82–0.97
Cervical	87	6.8	1.42 (0.73)	1.33	0.38 (0.52)	0.17	1.04 (0.83)	1.00	0.87–1.22	0.83–1.17
Non-vestibular	55	4.3	1.54 (0.76)	1.50	0.67 (0.91)	0.33	0.87 (1.24)	1.17	0.54–1.21	0.67–1.48
Unspecified	359	28.2	1.38 (0.66)	1.33	0.54 (0.74)	0.33	0.84 (0.89)	0.83	0.75–0.93	0.83–1.00
All patients	1274^b^	100	1.46 (0.69)	1.33	0.57 (0.75)	0.33	0.89 (0.97)	0.93	0.83–0.94	0.83–1.00

^a^95 % confidence intervals refer to the mean and median changes from baseline

^b^Data for one patient not available

The single vertigo symptoms most often reported as ‘severe’ or ‘very severe’ prior to the treatment were rotary sensation (43.7 %), dysstasia and walking unsteadiness (37.1 %) and staggering (30.6 %). At the end of observation, these symptoms were either disappeared or rated as ‘mild’ by 78.4 %, 64.6 %, respectively 69.0 % of the same patients.

Vertigo of peripheral or combined central-peripheral origin had been diagnosed in more than half of the study participants, whereas the type of vertigo could not be specified in 28 % of the patients. With respect to each type of vertigo, the reduction of the MVS was statistically significant (Wilcoxon signed rank test: *p* < 0.001); the mean and median reductions from baseline together with their 95 % CI are given in Table 4.

The mean score of the four vegetative concomitant symptoms nausea, vomiting, sweating and tachycardia decreased from 0.85 ± 0.80 to 0.37 ± 0.69, corresponding to a significant reduction by 56.5 % (0.48 ± 0.99; CI95 %: 0.42–0.53; *p* < 0.001). During the interim visit *(n* = 424), the reduction of the vegetative symptoms by 30.8 % (0.32 ± 0.96; CI95 %: 0.23–0.41) was already statistically significant *(p* < 0.001).

**Fig. 2:**
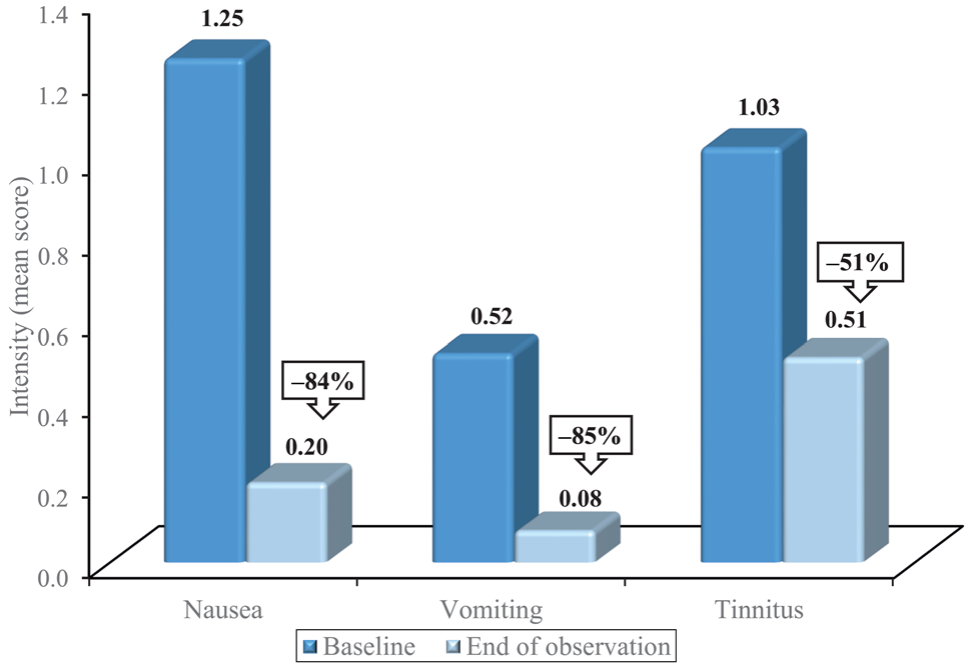
Reduction of the concomitant symptoms nausea, vomiting and tinnitus in the course of treatment with the fixed-combination preparation of cinnarizine 20 mg and dimenhydrinate 40 mg. All reductions in intensity were significant (*p* < 0.001)

Nausea and vomiting in particular, both frequently associated with vertigo, improved by 84 % and 85 %, respectively in the course of treatment. At baseline, nausea was reported by 832 (65.3 %) of all the patients. The mean value decreased from 1.25 ± 1.16 to 0.20 ± 0.44 (Fig. 2), which corresponds to a reduction by 84 % (1.05 ± 1.07; CI95 %: 0.14–0.26). Regarding only those 227 patients who described either ‘severe’ *(n* = 165) or ‘very severe’ *(n* = 62) nausea at baseline, 197 (86.8 %) reported either no *(n* = 118) or only ‘mild’ nausea *(n* = 79) at the end of observation.

A total of 404 patients (31.7 %) suffered from vomiting at baseline. The mean value improved by 85 % from 0.52 ± 0.91 to 0.08 ± 0.29 (reduction by 0.44 ± 0.87; CI95 %: 0.06–0.10; Fig. 2). In those 76 patients who initially reported the intensity of vomiting as ‘severe’ *(n* = 55) or ‘very severe’ *(n* = 21), the symptom was either no longer present *(n* = 52) or of only mild extent *(n* = 19) at the end of observation, which means a distinct improvement in 93.4 % of the patients.

Tinnitus was initially reported by 666 patients (52.2 %), and the mean value improved by 51 % from 1.03 ± 1.21 to 0.51 ± 0.73 (reduction by 0.52 ± 0.86; CI95 %: 0.47–0.55; Fig. 2). In those 213 patients who rated the intensity of tinnitus at baseline as ‘severe’ *(n* = 150) or ‘very severe’ *(n* = 63), the symptom was either no longer present *(n* = 28) or of only mild extent *(n* = 83) at the end of observation, corresponding to a distinct improvement in more than half of the patients.

On the occasion of the final consultation, the overall (global) efficacy was assessed by the physician using a 5-point verbal rating scale. After treatment with the fixed-combination preparation, the health condition was rated as either ‘very much improved’ in 40 % or ‘much improved’ in 45 % of the patients; ‘slightly improved’ and ‘not improved’ were reported for 13 and 2 % of the patients, respectively, whereas no deterioration of the health condition occurred in any patient.

In total, 58 nonserious AEs have been reported by 53 patients (4.2 %), 51 of which were judged as ADRs by the physician. The majority of ADRs were classified according to System Organ Class (SOC) (MedDRA^®^) as gastrointestinal disorders *(n* = 25; reported by 1.8 % of all the patients) or nervous system disorders *(n* = 15; 1.2 % of all the patients). The most commonly reported ADRs were somnolence/tiredness *(n* = 8), dry mouth *(n* = 8), nausea *(n* = 6) and headache *(n* = 6). All 51 ADRs were characterised as nonserious and the majority was of mild (45.1 %) or moderate (23.5 %) intensity. Moreover, the fixed-combination preparation did not show a clinically relevant effect on blood pressure. The physician’s assessment of tolerability of the treatment by means of a 4-point verbal rating scale was as follows: ‘very good’ in 843 (68.5 %), ‘good’ in 366 (29.7 %), ‘moderate’ in 15 (1.2 %) and ‘poor’ in 7 (0.6 %) out of 1231 patients.

## Discussion

The present noninterventional study shows that the low-dose fixed combination of cinnarizine and dimenhydrinate, as used under real life conditions, is effective at reducing both vertigo and frequently associated concomitant symptoms such as nausea and vomiting in patients suffering from vertigo of various origins. The study population largely mirrored a representative picture of patients with vertigo seen in daily medical practice. Particularly with respect to age and gender, the enrolled patients represented a typical vertigo population with a mean age of 61 years; the majority of patients being women [18].

The complexity of the vestibular system and limited availability of sophisticated diagnostic equipment often make it difficult or even impossible to establish an exact diagnosis, especially within the primary and secondary care setting. However, vertigo can impose considerable limitations on the patients’ ability to cope with daily activities, reduce quality of life and could become chronic if left untreated. Therefore, the authors believe that a rapid and effective symptomatic treatment is not only acceptable but in fact can be generally recommended. For this purpose, the fixed combination of cinnarizine and dimenhydrinate is particularly suited because of its broad range of efficacy in the treatment of vertigo. Due to the dual mode of action, with cinnarizine mainly acting on the peripheral vestibular system and dimenhydrinate on the central vestibular system, the combination preparation is well established as first-line treatment of vertigo in Germany and numerous other countries.

The broad spectrum of patients with vestibular (central, peripheral or combined central-peripheral) vertigo, as well as with vertigo of non-vestibular or unspecified origin included in this noninterventional study largely reflects the routine conditions in daily medical practice. Moreover, the recommended standard dosage of 3 × 1 tablet/day of the combination preparation had been prescribed to the vast majority of the patients (89 %), typically for the duration of around 6–8 weeks.

A validated composite score comprising six single vertigo symptoms (MVS) was considered the most appropriate approach to measure the patients’ outcome and due to its easy use, maximum assessment compliance could be expected.

Independent of the type of vertigo, treatment with the fixed-combination product led to a distinct overall reduction of vertigo symptoms by 61 %, with 22 % of all the patients being completely symptom-free at the end of observation. Even in patients suffering from vertigo of non-vestibular or unknown origin, the symptoms improved significantly. These results indicate that the fixed combination was effective in the symptomatic treatment of vertigo of various origins. Furthermore, the overall therapeutic success is adequately reflected in the physician’s global efficacy rating, where the vertigo complaints have been assessed as either ‘very much improved’ or ‘much improved’ in 85 % of the patients. This general judgement of efficacy might have been positively influenced by the distinct improvement of various concomitant symptoms, in particular nausea and vomiting, both frequently associated with vertigo and significantly reduced by 84 % and 85 %, respectively in the course of treatment.

All in all, the fixed-combination preparation showed the effectiveness which was to be expected from the findings of numerous controlled clinical trials. However, due to the noninterventional, open-label design, the present study has a number of limitations. These comprise—among others—the heterogeneity of the diagnoses with inclusion of patients suffering from different kinds of vertigo, and in particular the lack of a control group. Therefore, conclusions concerning the favourable efficacy outcomes should be made with caution. Moreover, the susceptibility of vertigo symptoms to placebo effects must be adequately taken into account when interpreting the efficacy results. Patients suffering from vertigo for a longer period of time, as was the case for many patients participating in this study, and who might have seen several other doctors before, generally feel a higher degree of discomfort and thus may be more susceptible to spontaneous healing; this is especially true in a study setting, where participants receive more attention than usual, which may contribute to create higher beliefs and expectations of improvement. In a previously conducted randomised, double-blind, active- and placebo-controlled study [13], the same MVS as used in the present noninterventional study showed for the combination preparation a mean reduction from 1.98 at baseline to 0.47 after 4-week treatment (− 1.51) as compared to a mean reduction from 1.91 to 1.08 (− 0.83) in the placebo group (unpublished results). The results showed that the combination preparation was nearly twice as effective as placebo in reducing vertigo symptoms; the difference was statistically significant (*p* < 0.001).

The safety evaluation showed a low rate of adverse reactions, mostly of mild or moderate intensity, all of them nonserious and without revealing any new or hitherto unknown side effects. The very good safety profile of the fixed-combination preparation is further reflected in a low dropout rate (one third because of complete remission of vertigo) and the physicians’ favourable rating of overall tolerability.

In conclusion, this noninterventional study demonstrates the favourable antivertiginous efficacy and safety profile of the fixed combination of cinnarizine and dimenhydrinate in daily medical practice. The study corroborates previous findings of numerous interventional, randomised, double-blind, controlled clinical trials [9–17] and confirms their validity in a setting representative of clinical routine. Because of its dual mode of action and synergistic effect, with cinnarizine acting as calcium antagonist and dimenhydrinate as antihistamine, the fixed-combination product effectively reduces vertigo and associated symptoms caused by a great variety of underlying vestibular or non-vestibular disorders. Especially in the primary and secondary care setting, where an exact diagnosis and subsequent causal treatment is quite often not possible, the combination preparation provides immediate symptomatic relief and is therefore considered as first-line treatment for patients with vertigo of various origins.
